# Modulating Transcriptional Regulation of Plant Biomass Degrading Enzyme Networks for Rational Design of Industrial Fungal Strains

**DOI:** 10.3389/fbioe.2018.00133

**Published:** 2018-09-25

**Authors:** Ebru Alazi, Arthur F. J. Ram

**Affiliations:** Molecular Microbiology and Biotechnology, Institute of Biology Leiden, Leiden University, Leiden, Netherlands

**Keywords:** CAZyme, plant biomass degradation, strain design, industrial fungi, transcriptional regulation, overexpression of transcription factors, constitutively active transcription factors, inducer accumulation

## Abstract

Filamentous fungi are the most important microorganisms for the industrial production of plant polysaccharide degrading enzymes due to their unique ability to secrete these proteins efficiently. These carbohydrate active enzymes (CAZymes) are utilized industrially for the hydrolysis of plant biomass for the subsequent production of biofuels and high-value biochemicals. The expression of the genes encoding plant biomass degrading enzymes is tightly controlled. Naturally, large amounts of CAZymes are produced and secreted only in the presence of the plant polysaccharide they specifically act on. The signal to produce is conveyed via so-called inducer molecules which are di- or mono-saccharides (or derivatives thereof) released from the specific plant polysaccharides. The presence of the inducer results in the activation of a substrate-specific transcription factor (TF), which is required not only for the controlled expression of the genes encoding the CAZymes, but often also for the regulation of the expression of the genes encoding sugar transporters and catabolic pathway enzymes needed to utilize the released monosaccharide. Over the years, several substrate-specific TFs involved in the degradation of cellulose, hemicellulose, pectin, starch and inulin have been identified in several fungal species and systems biology approaches have made it possible to uncover the enzyme networks controlled by these TFs. The requirement for specific inducers for TF activation and subsequently the expression of particular enzyme networks determines the choice of feedstock to produce enzyme cocktails for industrial use. It also results in batch-to-batch variation in the composition and amounts of enzymes due to variations in sugar composition and polysaccharide decorations of the feedstock which hampers the use of cheap feedstocks for constant quality of enzyme cocktails. It is therefore of industrial interest to produce specific enzyme cocktails constitutively and independently of inducers. In this review, we focus on the methods to modulate TF activities for inducer-independent production of CAZymes and highlight various approaches that are used to construct strains displaying constitutive expression of plant biomass degrading enzyme networks. These approaches and combinations thereof are also used to construct strains displaying increased expression of CAZymes under inducing conditions, and make it possible to design strains in which different enzyme mixtures are simultaneously produced independently of the carbon source.

## Introduction

Plant biomass is the most abundant renewable carbon source in the world and represents the major natural substrate for fungi (Kowalczyk et al., 2014). Plant biomass mainly consists of plant cell wall material, which contains the polysaccharides, i.e., cellulose, hemicellulose and pectin, lignin, and structural proteins (Loqué et al., 2015). Cellulose, the most abundant plant cell wall polysaccharide, is a linear chain of glucose (Kolpak and Blackwell, 1976). Hemicelluloses are complex heteropolysaccharides with xylan- (linear chain of xylose), glucan- (linear chain of glucose), glucomannan- (linear chain of glucose and mannose), or mannan- (linear chain of mannose) backbones, with several different types of monomers attached to the backbone to give e.g., arabinoxylan or xyloglucan (Scheller and Ulvskov, 2010). Pectins are other complex polysaccharides containing D-galacturonic acid (GA) as the main sugar acid in their backbones. Polygalacturonic acid (PGA) is a linear chain of GA and the most abundant pectin substructure. Other pectin substructures are rhamnogalacturonan I (linear chain of alternating GA and rhamnose residues), rhamnogalacturonan II and xylogalacturonan, and contain several different types of monomers or polymers attached to their backbones (Caffall and Mohnen, 2009). Plant biomass also contains the plant cell storage polysaccharides starch, a linear (amylose) or branched (amylopectin) polymer of glucose, and inulin, a polymer of fructose residues with a terminal glucose residue (Gidley, 2001; Ritsema and Smeekens, 2003).

Polysaccharides present in plant biomass are the target of the degrading enzymes secreted abundantly by filamentous fungi. Fungal carbohydrate active enzymes (CAZymes) are also utilized industrially for the hydrolysis of plant biomass for the subsequent production of mainly bioethanol, and high-value biochemicals (Kowalczyk et al., 2014; Gupta et al., 2016; Benocci et al., 2017). Plant biomass degrading enzymes are classified into families based on their sequence, such as glycoside hydrolases, polysaccharide lyases and carbohydrate esterases, abbreviated as GH, PL and CE, respectively (Carbohydrate Active Enzymes database, http://www.cazy.org/) (Lombard et al., 2014). Filamentous fungi secrete large amounts of CAZymes only in the presence of the plant polysaccharide they specifically act on. For a review on the substrate specificity of CAZymes we refer to Kowalczyk et al. (2014) and de Vries et al. (2017). The signal for tailor-made production of CAZymes is conveyed via so-called inducer molecules which are di- or mono-saccharides (or derivatives thereof) released from the specific plant polysaccharides. The presence of the inducer results in the activation of a substrate-specific transcription factor (TF), which is required for the controlled expression of the genes encoding not only the CAZymes, but usually also the transporters and catabolic pathway enzymes needed to utilize the released monosaccharide (Culleton et al., 2013; Benocci et al., 2017).

Several substrate-specific TFs involved in plant biomass degradation have been identified in filamentous fungal species. For a review on the TFs involved in plant biomass degradation we refer to Huberman et al. (2016) and Benocci et al. (2017). For example, it has been shown that induction of expression of the genes encoding cellulases when cellulose is present requires the presence of the transcriptional activators Clr-1 and Clr-2 in *Neurospora crassa*, ClrB in *Aspergillus nidulans* and *Penicillium oxalicum*, ManR in *A. oryzae* and Xyr1 in *Trichoderma reesei* (Stricker et al., 2006; Coradetti et al., 2012; Ogawa et al., 2013; Li et al., 2015). Clr-2, ClrB and ManR are orthologs (Kunitake and Kobayashi, 2017), while Xyr1 shows significant sequence similarity to the TFs involved in xylan degradation, XlnR in *A. niger* and Xlr-1 in *N. crassa* (Rauscher et al., 2006). The enzyme families controlled by orthologous transcription factors can vary. Although Xyr1 in *T. reesei* is essential for the expression of cellulases and xylanases, Xyr1 orthologs (named XlnR or Xlr-1 in other species) are essential only for the expression of xylanases in filamentous fungal species, such as *A. oryzae* (on xylose), *A. niger* (on xylan), *A. nidulans* (on xylan), *Talaromyces cellulolyticus* (on xylan), *P. oxalicum* (on cellulose), *N. crassa* (on xylan), *Fusarium oxysporum* (on xylan or wheat cell walls), and *Myceliophthora thermophila* (van Peij et al., 1998; Marui et al., 2002b; Stricker et al., 2006; Brunner et al., 2007; Tamayo et al., 2008; Sun et al., 2012; Fujii et al., 2014; Li et al., 2015; Wang et al., 2015). In *A. oryzae, A. niger, T. cellulolyticus*, and *P. oxalicum*, contribution of XlnR to the expression of cellulases during growth on cellulose and/or xylose/xylan has also been reported (Gielkens et al., 1999; Marui et al., 2002; Li et al., 2015; Okuda et al., 2016). Arabinan, present in the side chains of some form of hemicellulose, such as arabinogalactan, or some pectins, is degraded via CAZymes that are transcriptionally regulated by AraR in *A. niger* (Battaglia et al., 2011; Kowalczyk et al., 2017). Arabinan degradation in *T. reesei* is partially regulated via Xyr1 (Akel et al., 2009) and via Ara1 (Benocci et al., 2018). The TF GaaR, required for the expression of CAZymes degrading pectin, especially PGA, has been identified in both *Botrytis cinerea* and *A. niger* (Alazi et al., 2016; Zhang et al., 2016). Recently, Pdr-1 in *N. cr*assa was described as a TF required for the expression of the genes encoding CAZymes that degrade several pectin substructures including PGA and rhamnogalacturonan I (Thieme et al., 2017). Transcriptional control of the genes involved in starch degradation is conducted by AmyR in *A. niger* and *A. nidulans* (Tani et al., 2001; vanKuyk et al., 2012).

The transcriptional activators involved in plant biomass degradation mentioned above were identified either via classical approaches, such as mutant complementation (*xlnR* in *A. niger*) and gene cloning (*xlnR* and *amyR* in *A. nidulans* and *amyR A. niger*), or via post-genomic approaches, such as yeast one-hybrid screening (*gaaR* in *B. cinerea*), screening TF deletion mutant collections (*pdr-1* and *clr-2* in *N. crassa, manR* in *A. oryzae*, and *clrB* and *xlnR* in *P. oxalicum*), and also based on sequence similarity (*clrB* in *A. nidulans, araR* and *gaaR* in *A. niger, xyr1* in *T. reesei and M. thermophila*, and *xlnR* in *F. oxysporum, T. cellulolyticus* and *A. oryzae*) or their expression levels in the transcriptomics data (*xlr-1* in *N. crassa*) (van Peij et al., 1998; Tani et al., 2001; Marui et al., 2002b; Rauscher et al., 2006; Brunner et al., 2007; Battaglia et al., 2011; Coradetti et al., 2012; Ogawa et al., 2012; Sun et al., 2012; vanKuyk et al., 2012; Fujii et al., 2014; Li et al., 2015; Wang et al., 2015; Alazi et al., 2016; Zhang et al., 2016; Thieme et al., 2017). Positively acting TFs involved in controlling expression of plant cell wall degrading enzymes belong to the Zn_2_Cys_6_ type family of TFs. TFs belonging to this family are found specifically in fungi (both yeasts and filamentous fungi) and contain a DNA-binding domain with six cysteine residues bound to two zinc atoms, usually close to their NH_2_-terminal end. Most of the Zn_2_Cys_6_ type TFs also contain a fungal-specific TF domain, known as the middle homology region, with a proposed role in regulating the activity of the TF (MacPherson et al., 2006). Apart from activators several repressor proteins involved in plant biomass degradation have been identified including wide domain repressors, such as the carbon catabolite repressor protein CreA/Cre1, or substrate specific repressors which are discussed in paragraphs 1.2 and 2.3, respectively.

### Diversity in the control of transcription factor activities

The mechanisms by which the activity of TFs is regulated in response to stimuli can be diverse. Firstly, the protein level of the TFs might be controlled. This can be realized by regulation at the level of transcription, mRNA stability or protein stability resulting in low protein levels under non-inducing conditions and higher levels under inducing conditions. Secondly, the subcellular localization (nuclear import/export), DNA binding activity, and/or transcriptional activity of the TFs might be regulated by post-transcriptional modifications, such as phosphorylation, and/or via protein-protein or protein-metabolite interactions (MacPherson et al., 2006; Chang and Ehrlich, 2013; Tani et al., 2014). Finally, DNA site occupancy of TFs might depend on their cooperation or competition with other proteins and the chromatin accessibility (Granek and Clarke, 2005; Biggin, 2011).

Our knowledge about regulation of the activity of the TFs involved in biomass degradation is limited, yet growing. The amount of Clr-2 in *N. crassa* is regulated at the level of transcription by Clr-1, which gets activated and positively regulates *clr-2* expression only under inducing conditions (on insoluble crystalline cellulose Avicel or cellobiose). The expression of the *clr-2* ortholog in *A. nidulans, clrB*, on the other hand, is not drastically induced under inducing conditions (on Avicel) and the Clr-1 ortholog ClrA is not essential but only contributes to cellulase gene expression (Coradetti et al., 2012). This comparison indicates that activation mechanisms of even orthologous transcription factors differ between fungal species.

The expression of *xyr1* in *T. reesei* is subject to Cre1-dependent carbon catabolite repression (CCR) (see below) and induced on carbon sources inducing cellulase expression (e.g., lactose, sophorose, or cellulose), but not on carbon sources inducing xylanase expression (e.g., xylose) (Mach-Aigner et al., 2008; Portnoy et al., 2011a; Lichius et al., 2014). Xyr1 was shown to accumulate in the nucleus during growth on an inducing carbon source (i.e., sophorose or low concentration of xylose), whereas it is degraded in the nucleus during growth on a repressing carbon source (i.e., glucose or a high concentration of xylose). The increased amount of nuclear Xyr1 correlates with the increased expression level of cellulase gene *cbh1* on sophorose, and that of xylanase gene *xyn2* on a low concentration of xylose (Lichius et al., 2014). Similar to *T. reesei xyr1*, the expression of *xlnR* in *A. nidulans* and *P. oxalicum* is also subject to CreA-dependent CCR (see below), whereas *xlnR* in *A. niger* is not regulated at the level of transcription, but constitutively transcribed at low levels (Tamayo et al., 2008; Mach-Aigner et al., 2012; Li et al., 2015). Moreover, XlnR was found to be localized in the nucleus regardless of the presence of inducer in *A. niger* (Hasper, 2004). In *F. oxysporum, xlnR* is transcriptionally regulated by both CCR and induction on xylose/xylan (Calero-Nieto et al., 2007). XlnR in *A. oryzae* is reversibly phosphorylated in response to xylose, which does not affect its protein stability and correlates with the expression of XlnR target genes (Noguchi et al., 2011). Recently, Kunitake and Kobayashi proposed that a conserved sequence in XlnR is involved in the xylose-mediated phosphorylation of XlnR in *A. oryzae*, implying a conserved mechanism regulating XlnR activity among Ascomycete fungi. Moreover, the observation that XlnR in *A. oryzae* is not phosphorylated in response to cellobiose, but required for cellulase expression, indicates that its activity is regulated by a different mechanism on cellobiose (Noguchi et al., 2011; Kunitake and Kobayashi, 2017).

The activity of the GA-responsive transcriptional activator GaaR in *A. niger* is regulated by a different mechanism. It is suggested to be inhibited by the repressor protein GaaX via protein-protein interaction under non-inducing conditions. Under inducing conditions (in the presence of GA), the inducer is proposed to bind to GaaX, resulting in a free and active form of GaaR (Niu et al., 2017). The amount of GaaR is not significantly regulated at the level of transcription (Alazi et al., 2016, 2018). In addition, GaaR-eGFP was shown to be constitutively localized in the nucleus (Alazi et al., 2018). Transcription of *pdr-1* in *N. cr*assa is induced under inducing conditions (on rhamnose) and repressed under repressing conditions. Moreover, the activity of Pdr-1 is suggested to be post-transcriptionally regulated, as its nuclear accumulation and transcriptional activity requires the presence of rhamnose in a strain overexpressing *pdr-1* (Thieme et al., 2017).

The transcription of *amyR* is upregulated on inducing carbon sources (e.g., starch or maltose) and is subject to CreA-dependent CCR (see below) in *A. nidulans* (Tani et al., 2001). Further, AmyR was shown to localize in the nucleus in an inducer (i.e., isomaltose)-dependent manner and activate the expression of its target genes (Makita et al., 2009).

### The role of the carbon catabolite repressor in the production of plant biomass degrading enzymes

Apart from being upregulated under inducing conditions, the expression of CAZymes might also be controlled via CCR when a more energetically favorable carbon source, such as glucose, compared to plant biomass polysaccharides is available for fungi. The Cys_2_His_2_ type TF CreA/Cre1/Cre-1 (in *Aspergillus* species, *T. reesei*, and *N. crassa*, respectively) is the main transcriptional repressor contributing to CCR. Regulation of CreA activity has not been fully understood, but several studies have indicated that post-transcriptional modifications, such as ubiquitination and phosphorylation, affect CreA protein stability, subcellular localization and/or DNA binding activity (Cziferszky et al., 2002; Ries et al., 2016; see Adnan et al., 2017 for a recent review). CreA not only represses the expression of genes encoding CAZymes, but it might also repress the expression of some transcriptional activators, such as *clrB* and *xlnR* in *P. oxalicum, xyr1/xlnR* in *T. reesei, A. nidulans* and *F. oxysporum*, and *amyR* in *A. nidulans*, that are required for the expression of CAZymes (Tani et al., 2001; Calero-Nieto et al., 2007; Mach-Aigner et al., 2008; Tamayo et al., 2008; Li et al., 2015). Furthermore, CreA might repress the expression of CAZymes under high xylose concentrations, too, as was shown to be the case for XlnR and its target genes in *A. nidulans*, and for XlnR itself in *A. oryzae* (Tamayo et al., 2008; Ichinose et al., 2017). Elimination of CCR due to a lack of function of CreA and/or CreB, a ubiquitin-specific protease involved in CCR, or their orthologs has been reported to result in an increased expression of CAZymes degrading cellulose, hemicellulose, pectin or starch, under inducing and/or non-inducing conditions in filamentous fungi (Prathumpai et al., 2004; Mach-Aigner et al., 2008; Nakari-Setälä et al., 2009; Denton and Kelly, 2011; Sun and Glass, 2011; Fujii et al., 2013; Ichinose et al., 2014, 2017; Niu et al., 2015; Yang et al., 2015). However, even when CreA-dependent CCR is circumvented, the expression of CAZymes might require the presence of active transcriptional activators, which is normally achieved in the presence of inducing metabolites. For example, in a Cre1-negative *T. reesei* strain (Rut-C30), the full expression of Xyr1 target genes requires the presence of the inducer (i.e., xylose) (Mach-Aigner et al., 2008). Another study revealed that deletion of *cre1* in *T. reesei* results in elevated production of cellulases and xylanases under inducing (on lactose) and, to a lesser extent, under non-inducing conditions (on glucose) (Nakari-Setälä et al., 2009). Genome-wide studies in *T. reesei* have also indicated that only a few of the cellulolytic genes were upregulated in a Δ*cre1* strain during growth on glucose (Portnoy et al., 2011b; Antoniêto et al., 2014). This indicates that the majority of these cellulolytic genes are not induced simply by derepression, but require additional inducing condition for expression. Similarly, although the expression of pectinases is subject to CreA-dependent CCR in *A. niger*, deletion of *creA* is not sufficient for an increased production of polygalacturonases under non-inducing conditions. Polygalacturonases are only produced in the presence of the inducer (i.e., GA) or when *gaaX* is deleted, showing that GA-responsive gene expression requires the presence of active GaaR relieved from GaaX inhibition even in a Δ*creA* strain (Niu et al., 2015, 2017).

## Approaches for increased or constitutive expression of plant biomass degrading enzymes by modulating their transcriptional regulation

Plant biomass with varying sugar composition present in waste streams from agriculture, forestry and food industries represent a cheap, sustainable and renewable feedstock for the production of the CAZymes, and subsequently, valuable chemicals (Sweeney and Xu, 2012; Meyer et al., 2015). However, the composition of the enzyme cocktail will vary because of variation in the composition of the plant biomass. It is therefore of industrial interest to produce specific fungal enzyme cocktails constitutively, independently of inducers and the substrate used. Fungal production strains, such as *A. niger* CBS513.88, *T. reesei* Rut-C30, and *P. oxalicum* JU-A10-T, have been for a long time improved via multiple rounds of classical mutagenesis and screening approaches that can be time-consuming (Montenecourt and Eveleigh, 1979; Pel et al., 2007; Fang et al., 2010; van Hanh et al., 2010; Ho and Ho, 2015; Yao et al., 2015). However, emergence of the -omics era (genomics, transcriptomics etc.) and advances in recombinant technologies allow nowadays efficient strain improvement via genetic engineering approaches with minimal alterations in the genome (Meyer et al., 2010; Liu et al., 2013). In the remaining part of this review, we discuss the approaches to modulate transcriptional regulation in order to rationally design fungal strains with increased or constitutive production of plant biomass degrading enzymes, examples of which are given in Table 1. As will become clear in the following sections, the success of the approach used highly depends on the mechanism regulating the activity of the targeted TF.

**Table 1:** Examples of rational design of industrial fungal strains with increased or constitutive production of CAZymes.

	**Approach**	**CAZyme**	**Carbon source tested (Inducing/non-inducing)**	**References**
Ace1-*T. reesei*	Deletion of *ace1*	Cellulases, Xylanases	Inducing	Aro et al., 2003
AmyR-*A. nidulans*	Constitutive activation in AmyR_1−514_	Amylases	Inducing and non-inducing	Makita et al., 2009
AmyR-*A. niger*	Overexpression of *amyR*	Cellulases, hemicellulases, amylases	Inducing	vanKuyk et al., 2012
Clr-2-*N. crassa*	Overexpression of *clr-2*	Cellulases	Inducing and non-inducing	Coradetti et al., 2013
ClrB-*A. nidulans*	Overexpression of *clrB*	Cellulases	Inducing	Coradetti et al., 2013
ClrB (and CreA) (and β-glucosidase)-*P. oxalicum*	Overexpression of *clrB* (and deletion/a lack of function of *creA*) (and deletion of *bgl2*)	Cellulases (and hemicellulases)	Inducing (and non-inducing)	Chen et al., 2013; Li et al., 2015; Yao et al., 2015
CreA/CreB or their orthologs-see the text	Elimination of CCR by deletion/a lack of function of *creA*/*creB*	Cellulases, hemicellulases, pectinases, amylases	Inducing and/or non-inducing	See the text
GA catabolic pathway-*A. niger*	Deletion of *gaaC*	Pectinases	Inducing	Alazi et al., 2018
GaaR (and CreA)-*A. niger*	Overexpression of *gaaR* (and deletion/a lack of function of *creA*)	Pectinases	Inducing and non-inducing	Alazi et al., 2017
	Constitutive activation in GaaR^W361R^	Pectinases	non-inducing	Alazi et al., in press
GaaX-*A. niger*	Deletion of *gaaX*	Pectinases	Non-inducing	Niu et al., 2017
GCN5-related N-acetyltransferase -*T. reesei*	Overexpression of gene 123668	Cellulases	Inducing	Häkkinen et al., 2014
Hcr-1-*N. crassa*	Deletion of *hcr-1*	Xylanases	Inducing	Li et al., 2014
Lae1-*T. reesei*	Overexpression of *lae1*	Cellulases	Inducing	Seiboth et al., 2012b
ManR-*A. oryzae*	Overexpression of *manR*	Cellulases, Mannanases	Inducing	Ogawa et al., 2012, 2013
Mhr1-*M. thermophila*	Gene silencing of *mhr1*	Cellulases, Xylanases	Inducing	Wang et al., 2018
Pdr-1-*N. crassa*	Overexpression of *pdr-1*	Pectinases	Inducing	Thieme et al., 2017
Rce1-*T. reesei*	Deletion of *rce1*	Cellulases	Inducing	Cao et al., 2017
Synthetic TF-*P. oxalicum*	Overexpression of *cx^*c*^-s*	Cellulases, Xylanases	Inducing and non-inducing	Gao et al., 2017a
Synthetic TF-*T. reesei*	Overexpression of *xyr1-cre1_*b*_*	Cellulases, Xylanases	Non-inducing	Zhang et al., 2017
SxlR-*T. reesei*	Deletion of *sxlR*	Xylanases	Inducing	Liu et al., 2017
XlnR-*A. nidulans*	Overexpression of *xlnR*	Xylanases	Inducing	Tamayo et al., 2008
XlnR-*A. niger*	Overexpression of *xlnR*	Cellulases, Xylanases	Inducing	Gielkens et al., 1999
	Constitutive activation in XlnR^V756F^/XlnR^L668Stop^	Xylanases	Inducing and non-inducing	Hasper, 2004; Hasper et al., 2004
XlnR-*A. oryzae*	Overexpression of *xlnR*	Cellulases, Xylanases	Inducing	Marui et al., 2002; Noguchi et al., 2009
XlnR-*F. oxysporum*	Overexpression of *xlnR*	Xylanases	Inducing	Calero-Nieto et al., 2007
XlnR-*P. oxalicum*	Constitutive activation in XlnR^A871V^	Cellulases, hemicellulases	Inducing	Gao et al., 2017a
XlnR-*T. cellulolyticus*	Overexpression of *xlnR*	Cellulases	Inducing	Okuda et al., 2016
Xlr-1-*N. crassa*	Constitutive activation in Xlr-1^A828V^	Xylanases	Inducing and non-inducing	Craig et al., 2015
Xpp1-*T. reesei*	Deletion of *xpp1*	Xylanases	Inducing	Derntl et al., 2015
Xylose/arabinose catabolic pathway-*T. reesei*	Deletion of xylose/arabinose catabolic pathway genes	Xylanases	Inducing	Herold et al., 2013
Xyr1-*M. thermophila*	Overexpression of *xyr1*	Xylanases	Inducing and non-inducing	Wang et al., 2015
Xyr1-*T. reesei*	Overexpression of *xyr1* (and gene silencing of *ace1* in RUT-C30)	Cellulases (and xylanases)	Inducing and non-inducing	Wang et al., 2013; Lv et al., 2015
	Constitutive activation in Xyr1^A824V^	Cellulases, Xylanases	Inducing and non-inducing	Derntl et al., 2013
β-glucosidases-*N. crassa*	Deletion of *gh1-1, gh3-3* and *gh3-4* (and deletion/a lack of function of *cre-1)*	Cellulases	Inducing	Znameroski et al., 2012

### Overexpression of TFs

Increasing the amount of a TF at the level of transcription can be achieved by expressing multiple copies of the TF via its endogenous promoter, or (multiple copies of) the TF via a strong inducible/constitutive promoter. Overexpression of several TFs in *Saccharomyces cerevisiae* has been shown to result in an increased expression of the TF target genes, even under non-inducing conditions (Chua et al., 2006).

Inducer-independent production of cellulases in *N. crassa* was achieved by constitutively overexpressing *clr-2* via the *ccg-1* promoter (Coradetti et al., 2013). While deletion of *clr-2* resulted in a drastic decrease in the expression of most of the genes encoding cellulases under inducing conditions [i.e., on insoluble crystalline cellulose (Avicel)], overexpression of *clr-2* resulted in an increased expression of cellulases under inducing conditions and constitutive expression under non-inducing conditions. Expression of the genes encoding cellulases was higher under starvation (no carbon) conditions than on sucrose highlighting the effect of CCR on cellulase encoding genes under repressing conditions (i.e., on sucrose) even when *clr-2* was overexpressed (Coradetti et al., 2013).

Unlike overexpression of *clr-2* in *N. crassa*, overexpression of the *clr-2* ortholog *clrB* in *A. nidulans* or in *P. oxalicum*, both via the constitutive *A. nidulans gpdA* promoter, did not result in an increased expression of CAZymes encoding genes under non-inducing conditions, but only under inducing conditions (on Avicel or cellulose, respectively) (Coradetti et al., 2013; Li et al., 2015). It was shown that deletion of *creA* in combination with *clrB* overexpression allows increased expression of cellulases under non-inducing conditions in *P. oxalicum*, indicating that the strong CreA-dependent repression on cellulase genes overrules ClrB-dependent induction (Li et al., 2015).

ManR in *A. oryzae* is the *N. crassa clr-2* ortholog, regulating the expression of genes encoding cellulose and hemicellulose (including mannan, but not xylan) degrading enzymes in *A. oryzae* (Ogawa et al., 2012, 2013). Overexpression of ManR via the *tef1* promoter resulted in an increased expression of cellulases and hemicellulases involved in mannan degradation under inducing conditions (i.e., on Avicel and mannan, respectively) (Ogawa et al., 2012, 2013). The effect of overexpression of ManR under non-inducing conditions has not been reported.

As mentioned before, in *T. reesei*, Xyr1 controls the transcriptional regulation of both cellulase encoding and xylanase encoding genes. Expression of *xyr1* is subject to CCR and *xyr1* expression is upregulated on carbon sources inducing cellulase production. Overexpression of *xyr1* via the *tcu1* promoter resulted in an inducer-independent production of cellulases, even under repressing conditions (i.e., on glucose) (Lv et al., 2015). All reported *T. reesei* Xyr1 orthologs in other filamentous fungal species regulate mainly the transcription of hemicellulase encoding genes and contribute less to the transcriptional regulation of cellulase encoding genes. Overexpression of *xlnR* via *gpdA* promoter in *T. cellulolyticus* resulted in an increased production of cellulases on cellulose, but not that of xylanases (Okuda et al., 2016). In *A. niger*, increased expression of cellulases and xylanases on xylose was observed in a strain carrying multiple copies of *xlnR* (Gielkens et al., 1999). Similarly, an *A. oryzae* strain overexpressing *xlnR* via the *tef1* promoter showed an increased production of both cellulases and xylanases when grown on carbon sources known to induce cellulase (i.e., Avicel or cellobiose) or xylanases (i.e., xylose or xylan) production (Marui et al., 2002; Noguchi et al., 2009).

The studies mentioned above reported on the increased expression of xylanases in fungal strains overexpressing *xlnR* or its orthologs under inducing conditions. Only a few studies have reported the effect of overexpression of *xlnR* on the expression of xylanases under non-inducing conditions. For instance, *xlnR* overexpression via the constitutive *gpdA* promoter in *A. nidulans* or in *F. oxysporum* yields to increased production of xylanases encoding genes only in the presence of inducing carbon sources (e.g., xylose or xylan) (Calero-Nieto et al., 2007; Tamayo et al., 2008). It was also reported that when GFP-XlnR is overproduced in *A. oryzae*, it localizes constitutively in the nucleus, but its target genes are expressed only in the presence of xylose (Noguchi et al., 2011). These results indicate that XlnR in these organisms gets activated by an inducer-mediated post-transcriptional mechanism and that XlnR activation under non-inducing conditions cannot simply be achieved by overexpression. In *M. thermophila*, overexpression of *xyr1* via the *pdc* promoter resulted in an increased expression of xylanases under both inducing (i.e., corncob) and non-inducing conditions (i.e., glucose) (Wang et al., 2015). This indicates that the mechanism of Xyr1 activation in *M. thermophila* differ from other filamentous fungi.

Another example of inducer-independent production of CAZymes by overexpression of TFs was given by a recent study *in A. niger*. Here it was shown that overexpression of *gaaR* in *A. niger* via the *A. nidulans gpdA* promoter leads to constitutive expression of the genes encoding pectinases, as well as GA transporters and GA catabolic pathway enzymes (Alazi et al., 2018). Deletion of *creA* further enhanced pectinase production under mildly repressing conditions (i.e., on fructose) indicating competing roles of GaaR and CreA to control the expression of GA-induced genes (Alazi et al., 2018). The effect of overexpression of *gaaR* on the expression of its target genes under non-inducing conditions is likely caused by its specific activation mechanism. Regulation of the activity of GaaR includes a specific repressor protein (GaaX) (Niu et al., 2017). It has been proposed that under non-inducing conditions the activity of GaaR is controlled through direct interaction by GaaX which prevents GaaR to be active (Niu et al., 2017). Modulating the amount of GaaR by overexpression affects the balance of GaaR-GaaX and results in the presence of uncomplexed active GaaR even under non-inducing conditions (Alazi et al., 2018).

The regulation of GA-induced gene expression shows some striking similarities with the regulation of genes involved in quinic acid catabolism. Quinic acid is present as an aromatic compound in the plant cell and can be released from tannins, which are water-soluble polyphenols, by the action of tannases known to be secreted by filamentous fungi (Wagh, 2010). Similar to regulation of GA utilization in *A. niger*, regulation of quinic acid utilization in *A. nidulans* involves a Zn_2_Cys_6_ type transcriptional activator (QutA) and a repressor (QutR), and strains expressing multiple copies of *qutA* displayed constitutive expression of the genes encoding quinic acid catabolic pathway enzymes (Lamb et al., 1996). The QutR and GaaX repressor proteins are both multidomain proteins with sequence similarity to the three C-terminal domains of AROM, indicating that these repressors share a common evolutionary origin (Niu et al., 2017).

On the other hand, overexpression of *pdr-1*, the pectin degradation regulator in *N. crassa*, via the *gpd* promoter of *M. thermophila* resulted in elevated expression of its target genes only under inducing conditions (on rhamnose). Therefore, it was proposed that Pdr-1 activity is regulated post-transcriptionally in a manner depending on the presence of the inducer but not on the amount of Pdr-1 (Thieme et al., 2017).

An *A. niger* strain carrying multiple copies of *amyR* displayed increased expression of AmyR target genes, such as CAZymes acting on starch as well as on cellulose and hemicellulose, under inducing conditions (i.e., on maltose, starch, and low concentration of glucose), but not under non-inducing condition (vanKuyk et al., 2012). This result is in line with the observation that in *A. nidulans*, nuclear localization of AmyR and thereby activation of its target genes is inducer-dependent (Makita et al., 2009).

In conclusion, we have seen that overexpression of TFs involved in plant biomass degradation can result in increased expression of their target genes under inducing conditions. However, in most cases overexpression of TFs, such as XlnR, Pdr-1 and AmyR, does not result in inducer-independent expression of their target genes. In these cases it is likely that an inducer molecule is required to activate the TF. The exact activation mechanism of XlnR, Pdr-1, and AmyR are currently unknown, and could involve direct interaction of the TF with its inducer, or could be related to post-translational modifications connected to the presence of inducers. Overexpression of TFs like GaaR and QutA results in inducer-independent expression of GaaR and QutA target genes most likely by titrating away the corresponding repressor proteins (GaaX and QutR, respectively). This illustrates that different mechanisms controlling TF activities result in different outcomes of overexpression of TFs under non-inducing conditions.

### Constitutive activation of TFs

The activity of a TF can be controlled in different ways, including post-transcriptional modifications affecting its localization, DNA binding or interaction with repressor protein(s). Over the past 15 years, mutations resulting in changes in amino acid sequences and thereby in constitutively active TFs have been identified via classical mutagenesis and screening approaches, or via pre-designed amino acid substitutions, domain removal or protein truncation analyses.

The first reported constitutively active form of XlnR (XlnR^V756F^) was identified in *A. niger* via a forward genetic screen. Expression of *xlnR*^*V*756*F*^ resulted in a constitutive expression of xylanases even under repressing conditions (i.e., on fructose or glucose; Hasper, 2004; Hasper et al., 2004). Later, in *T. reesei*, a point mutation in Xyr1 (Xyr1^A824V^) introduced via UV mutagenesis was found to result in a constitutively active Xyr1 and constitutive expression of cellulases and xylanases even in the presence of a repressing carbon source (Derntl et al., 2013). In addition, overexpression of *xyr1*^*A*824*V*^ in *T. reesei* was shown to be more effective in increasing cellulase production than overexpression of *xyr1* under inducing conditions (e.g., Avicel) (Jiang et al., 2016). Both amino acids changes (V756F in XlnR and A824V in Xyr1) are located within the same predicted α-helix in the fungal-specific TF domain of XlnR/Xyr1 (Derntl et al., 2013). Although overexpression of *xlr-1* in *N. crassa* via the *ccg-1* promoter did not yield to constitutive expression of xylanases, overexpression of *xlr-1*^*A*828*V*^, which carries the homologous mutation as in *xyr1*^*A*824*V*^, resulted in a constitutive and increased production of xylanases under both inducing (on xylan) and non-inducing conditions (Craig et al., 2015). Similarly, overexpression of *xlnR* carrying the homologous point mutation (*xlnR*^*A*871*V*^) using the *PDE_02864* promoter in a *P. oxalicum* strain that lacks *creA* and overexpresses *clrB* (see above), enabled even more increased production of cellulases and xylanases under inducing conditions (on wheat bran) (Gao et al., 2017a).

Recently, via a forward genetic screen, several different point mutations throughout AraR were found to give rise to a constitutively active AraR and constitutive expression of AraR target genes (*abfA, abfB*, and *abfC*). Unlike the mutations in XlnR that gave rise to inducer-independent and constitutive expression of its target genes, the mutations in AraR lead to constitutive expression of AraR target genes only under de-repressed conditions. Deletion of *creA* improved the production of CAZymes that degrade arabinan to a large extent, indicating the strong CCR on the genes encoding these CAZymes under repressing conditions on fructose (Reijngoud et al., manuscript in preparation).

We recently conducted a large forward genetic screen for mutants with constitutive expression of pectinases (Niu et al., 2017). Apart from identifying GaaX as a repressor for GA-induced genes expression, it also brought about the identification of a constitutive allele of GaaR (GaaR^W361R^) resulting in constitutive expression of pectinases under even repressing conditions (on glucose or fructose). Within GaaR, W361 is situated in the fungal-specific TF domain and is highly conserved among *Aspergillus* species (Alazi et al., in press).

Deletion of the C-terminal regions of several TFs involved in plant biomass degradation was shown to result in constitutive activation of the TFs indicating that these C-terminal parts contain an inhibitory domain. For example, in *A. niger*, truncation of XlnR from L668 resulted in a constitutive expression of xylanases (Hasper et al., 2004), and truncation of AraR from P646 was reported to cause constitutive activation of AraR (Jiang et al., 2016). Expression of the C-terminally truncated AmyR_1−514_ and AmyR_1−511_ resulted in constitutive localization of AmyR in the nucleus both in *A. nidulans* and *A. oryzae*, respectively. However, while constitutive amylase production was observed in *A. nidulans* (Makita et al., 2009), loss of expression was observed in *A. oryzae* (Suzuki et al., 2015), indicating that the effect of the truncation can also be species-specific.

### Deletion or down-regulation of specific repressors

It has been shown that besides the general CCR, specific repressors might play a role in the transcriptional regulation of the genes encoding CAZymes. For instance, the Cys_2_His_2_ type TF Ace1 represses the expression of both cellulase and hemicellulase (i.e., xylanase) encoding genes, as well as the expression of *xyr1* in *T. reesei* (Aro et al., 2003; Wang et al., 2013). Furthermore, Ace1 was shown to compete with Xyr1 to bind to the same sequence in the promoter of the xylanase gene *xyn1* (Rauscher et al., 2006). Deletion of *ace1* in the wild type background resulted in an increased expression of the genes encoding cellulases and xylanases only under inducing conditions (on cellulose), and gene silencing of *ace1* led to an increase in constitutive expression of these genes when combined with the overexpression of *xyr1* via the *pdc* promoter in a Cre1-negative background (Rut-C30) (Aro et al., 2003; Wang et al., 2013). Recently, another repressor, the Zn_2_Cys_6_ type TF Rce1, was identified in *T. reesei* that is involved in the repression of the genes encoding cellulases, but not xylanases. It was shown that Rce1 is constitutively localized in the nucleus and competes with Xyr1 to bind to the same sequence in the promoter of the cellulase gene *cbh1* (Cao et al., 2017). Deletion of *rce1* resulted in an increased production of cellulases under inducing conditions (on cellulose) (Cao et al., 2017). Deletion of a basic helix-loop-helix TF *xpp1*, xylanase promoter-binding protein 1, in *T. reesei* resulted in an increased expression of hemicellulase (i.e., xylanase) encoding genes, but not cellulase encoding genes, at late stages of cultivation under inducing conditions (on xylan) (Derntl et al., 2015). However, Xpp1 was later described as a general regulator of both primary and secondary metabolism and therefore not a factor specifically controlling xylanases expression (Derntl et al., 2017). In addition, the Zn_2_Cys_6_ type transcriptional repressor SxlR was found to bind to the promoters of specific (GH11 family) xylanase genes, and deletion of *sxlR* resulted in an increased expression of these xylanase genes under inducing conditions (on Avicel, xylan or lactose) (Liu et al., 2017).

Similarly, the Cys_2_His_2_ type TF Hcr-1 in *N. crassa* was shown to repress the expression of the genes encoding xylanases, but not the ones encoding cellulases. A Δ*hcr-1* strain exhibited an increased xylanase production under inducing conditions (on xylan or Avicel) (Li et al., 2014).

Recently a new TF, MhR1, was identified in *M. thermophila* as the regulator of cellulase and xylanase genes. Gene silencing of *mhr1* resulted in an increased production of cellulases and xylanases, as well as increased expression of *xyr1* and the genes encoding cellulases under inducing conditions (on wheat straw) (Wang et al., 2018).

As mentioned before, the activity of GaaR in *A. niger* is inhibited by the repressor protein GaaX, the amount of which is regulated at the level of transcription by induction on GA. GaaX was proposed to bind to- and inhibit GaaR under non-inducing conditions, and bind to the inducer molecule and release GaaR under inducing conditions. Deletion of *gaaX* resulted in a constitutive expression of pectinases, providing additional evidence for the proposed model of regulation of GA-responsive gene expression in *A. niger* (Niu et al., 2017). Similarly, deletion of QutR, the repressor protein involved in controlling the expression of quinic acid-responsive genes, also results in constitutive expression of at least eight genes involved in quinic acid uptake and metabolism (Levett et al., 2000).

### Accumulation of inducers

The intracellular accumulation of inducers is another effective method to boost the production of CAZymes by fungi. Cellulase production by *N. crassa* is greatly induced on insoluble, crystalline cellulose (Avicel). However, cellulase production is not observed on cellobiose, which is the soluble degradation product of cellulose, possibly due to rapid action of β-glucosidases converting cellobiose to glucose and subsequent glucose-mediated CCR. As first shown in *N. crassa*, deletion of three genes encoding the major β-glucosidases (intracellular enzyme Gh1-1, and extracellular enzymes Gh3-3 and Gh3-4) disrupted the hydrolysis of the inducer, cellobiose, and resulted in higher levels of expression of cellulases on cellobiose compared to the wild type strain grown on cellobiose, and similar to the wild type strain grown on Avicel. Moreover, deletion of *cre-1* further increased cellulase production on cellobiose (Znameroski et al., 2012). Similarly, deletion of the major intracellular β-glucosidase *bgl2* in a carbon catabolite de-repressed (Δ*creA*) *P. oxalicum* strain that overexpresses *clrB* using the *A. nidulans gpdA* promoter, gave rise to higher levels of cellulase and hemicellulase (i.e., xylanase) production on cellulose compared to the wild type strain. These were similar to the levels of the industrial strain JU-A10-T grown on wheat bran (Yao et al., 2015). A similar effect of deleting *bgl2* on cellulase and hemicellulase production was observed in *P. oxalicum* grown on cellulose (Chen et al., 2013).

Expression of hemicellulases, specifically xylanases, in *T. reesei* is induced both on xylose and arabinose. Although the catabolic pathways assimilating xylose and arabinose are interconnected, the physiological inducers triggering hemicellulase production appear to be different and were suggested to be xylose and L-arabitol, respectively. Expression of xylanases increased dramatically in single or double xylose/arabinose catabolic pathway enzyme deletion mutants, indicating the effect of accumulation of physiological inducers in these mutants (Seiboth et al., 2012a; Herold et al., 2013).

One of the GA catabolic pathway intermediates, 2-keto-3-deoxy-L-galactonate, was recently shown to be the physiological inducer of the genes encoding pectinases in *A. niger*. It was demonstrated that deletion of *gaaC*, the gene encoding 2-keto-3-deoxy-L-galactonate aldolase, results in accumulation of 2-keto-3-deoxy-L-galactonate and thereby elevated expression levels of pectinase encoding genes (Alazi et al., 2018). Inducer molecules that are responsible to the activation of TFs represent therefore another interesting target to enhance expression of CAZymes by constructing strains in which the inducer accumulates either due to prevention of rapid hydrolysis of the inducer or due to inactivation of the metabolic genes that function downstream of the enzyme that forms the inducer.

## Future perspectives and concluding remarks

Increased expression of plant cell wall degrading enzymes can be achieved by overexpression of specific transcription factors or by identifying mutations in TFs leading to constitutive activation, and combinations thereof. It is also well established that CreA-dependent CCR has in many cases a negative effect on the production of enzymes and therefore CreA is an important target for improving enzyme production under both inducing and non-inducing conditions. Apart from the approaches described above in the previous paragraphs, modulating chromatin accessibility is yet an under-utilized approach to increase the expression of CAZyme encoding genes in filamentous fungi. Chromatin remodeling of the promoters of CAZyme encoding genes through the actions of the histone acetyltransferase Gcn5, the CCAAT-binding complex, and possibly the putative protein methyltransferase Lae1 is required for the full expression of these CAZymes in *T. reesei* (Zeilinger et al., 1998; Xin et al., 2013; Aghcheh and Kubicek, 2015; Li et al., 2016). Overexpression of a putative GCN5-related N-acetyltransferase (gene ID 123668) via the *A. nidulans gpdA* promoter or *lae1* via the *tef1* promoter resulted in an increased expression of cellulase encoding genes under inducing conditions (on lactose) (Seiboth et al., 2012b; Häkkinen et al., 2014). More recently, Cre1 was shown to be involved in chromatin accessibility of *xyr1* promoter (Mello-de-Sousa et al., 2016).

As our knowledge about regulation of TF activities accumulates, various possibilities for rational design of industrial fungal strains emerge, such as combinations of different approaches as already mentioned above, or the use of synthetic TFs. Recently, inducer-independent production of CAZymes by filamentous fungi was achieved via synthetic TFs. For instance, overexpression via *pdc1* promoter of a synthetic TF (*xyr1-cre1*_*b*_) consisting of Xyr1 DNA-binding and effector domains, and Cre1 DNA-binding domain, resulted in inducer-independent production of cellulases and hemicellulases (i.e., xylanases) in the Cre1-negative *T. reesei* strain Rut-C30 (Zhang et al., 2017). Another example of synthetic TFs (CX^C^-S) was shown in *P. oxalicum*, where the DNA-binding domain of the constitutively active XlnR^A871V^ was replaced with that of ClrB. This synthetic TF was overexpressed using the *A. nidulans gpdA* promoter, yielding to inducer-independent, but still glucose-repressed, expression of cellulases and xylanases (Gao et al., 2017a,b).

To conclude, fungal strains with increased or constitutive production of plant biomass degrading enzymes can be rationally designed by tuning the transcriptional regulatory systems. Mutations that lead to constitutive expression of enzymes are difficult to predict and so far only identified via genetic screens. Once identified, these mutations can be successfully transferred to industrial strains or related species. In this review, we also highlighted that regulation of activities of orthologues TFs, or the set of genes regulated by orthologues TFs might be species-specific. It is therefore important that detailed studies on how TFs are activated are performed not only in a single species, which is subsequently used as a blue print to predict the activation mechanism in other fungal species, but performed in several representative fungal species across the filamentous fungi.

## Author contributions

EA and AR designed the content of the manuscript and collected literature. EA wrote the manuscript and AR critically commented on the manuscript.

### Conflict of interest statement

The authors declare that the research was conducted in the absence of any commercial or financial relationships that could be construed as a potential conflict of interest.

## Abbreviations

CAZyme: carbohydrate active enzyme
GA: D-galacturonic acid
PGA: polygalacturonic acid
TF: transcription factor
CCR: carbon catabolite repression.
